# TGF-Beta Induced Key Genes of Osteogenic and Adipogenic Differentiation in Human Mesenchymal Stem Cells and MiRNA–mRNA Regulatory Networks

**DOI:** 10.3389/fgene.2021.759596

**Published:** 2021-11-25

**Authors:** Genfa Du, Xinyuan Cheng, Zhen Zhang, Linjing Han, Keliang Wu, Yongjun Li, Xiaosheng Lin

**Affiliations:** ^1^ Department of Orthopedics, Shenzhen Hospital of Integrated Traditional Chinese and Western Medicine, Guangzhou University of Chinese Medicine, Shenzhen, China; ^2^ The Fourth Clinical Medical College, Guangzhou University of Chinese Medicine, Shenzhen, China; ^3^ Department of Orthopedics, Shunde Hospital Guangzhou University of Chinese Medicine, Foshan, China

**Keywords:** TGF-beta, osteoporosis, obesity, osteogenesis, adipogenesis, mesenchymal stem cell

## Abstract

**Background:** The clinical efficacy of osteoporosis therapy is unsatisfactory. However, there is currently no gold standard for the treatment of osteoporosis. Recent studies have indicated that a switch from osteogenic to adipogenic differentiation in human bone marrow mesenchymal stem cells (hMSCs) induces osteoporosis. This study aimed to provide a more comprehensive understanding of the biological mechanisms involved in this process and to identify key genes involved in osteogenic and adipogenic differentiation in hMSCs to provide new insights for the prevention and treatment of osteoporosis.

**Methods:** Microarray and bioinformatics approaches were used to identify the differentially expressed genes (DEGs) involved in osteogenic and adipogenic differentiation, and the biological functions and pathways of these genes were analyzed. Hub genes were identified, and the miRNA–mRNA interaction networks of these hub genes were constructed.

**Results:** In an optimized microenvironment, transforming growth factor-beta (TGF-beta) could promote osteogenic differentiation and inhibit adipogenic differentiation of hMSCs. According to our study, 98 upregulated genes involved in osteogenic differentiation and 66 downregulated genes involved in adipogenic differentiation were identified, and associated biological functions and pathways were analyzed. Based on the protein–protein interaction (PPI) networks, the hub genes of the upregulated genes (CTGF, IGF1, BMP2, MMP13, TGFB3, MMP3, and SERPINE1) and the hub genes of the downregulated genes (PPARG, TIMP3, ANXA1, ADAMTS5, AGTR1, CXCL12, and CEBPA) were identified, and statistical analysis revealed significant differences. In addition, 36 miRNAs derived from the upregulated hub genes were screened, as were 17 miRNAs derived from the downregulated hub genes. Hub miRNAs (hsa-miR-27a/b-3p, hsa-miR-128-3p, hsa-miR-1-3p, hsa-miR-98-5p, and hsa-miR-130b-3p) coregulated both osteogenic and adipogenic differentiation factors.

**Conclusion:** The upregulated hub genes identified are potential targets for osteogenic differentiation in hMSCs, whereas the downregulated hub genes are potential targets for adipogenic differentiation. These hub genes and miRNAs play important roles in adipogenesis and osteogenesis of hMSCs. They may be related to the prevention and treatment not only of osteoporosis but also of obesity.

## Introduction

Osteoporosis is one of the most common chronic aged-related diseases in the world, and it is especially common in postmenopausal women (Zhi et al., 2021). It is characterized by loss of bone mass, degeneration of bone microstructure, and reduction of bone strength (Black and Roen, 2016). It is a highly prevalent disease that affects an estimated 200 million people worldwide (Vellucci et al., 2014). It has been reported that approximately 50% of women and 20% of men over the age of 50 years will have osteoporotic fractures in their remaining years (Sambrook and Cooper, 2006). This inevitably leads to higher mortality, high medical costs, and social burden. Notably, however, there is still no gold standard for the treatment of osteoporosis (McClung et al., 2019). Drugs such as bisphosphonates, calcitonin, and estrogen can delay the progression of osteoporosis (Chang et al., 2019; Eastell et al., 2019), but these drugs must be taken for a long time and may cause serious side effects (Ensrud and Crandall, 2017). Thus, it is very important to understand the pathogenesis of osteoporosis, and further investigation of novel osteoporosis targets is imperative.

Bone marrow mesenchymal stem cells (BMSCs) have the capacity to differentiate into many cell types, including osteoblasts and adipocytes (Yu et al., 2021), which are closely associated with osteoporosis (Haasters et al., 2014). Previous studies have indicated that the connection between fat and bone is a significant factor in the pathology of senile bone loss and that fat in bone marrow could be used as a diagnostic and therapeutic tool in osteoporosis (Duque, 2008). In recent years, there has been increasing interest in the interaction between fat and bone cells in bone marrow (van Zoelen et al., 2016). It has been reported that an imbalance between bone formation and bone loss occurs with aging and that the bone marrow component shifts to adipocytes, osteoclast activity enhances, and osteoblast function declines (Rosen and Bouxsein, 2006; Hu et al., 2018). Osteogenic differentiation is inhibited and bone formation is reduced, leading to bone that is filled with adipocytes instead of osteoblasts, thereby inducing osteoporosis (Rosen and Bouxsein, 2006; Hu et al., 2018). Therefore, there is a negative correlation between bone formation and fat accumulation in bone marrow (Souza et al., 2021). Although there is a competitive relationship between adipogenesis and osteogenesis during the differentiation of human BMSCs (hMSCs), the adipo-osteogenic signaling pathway could be altered to favor osteoblasts for the prevention of osteoporosis (Wu et al., 2020a). Notably, however, the specific mechanisms of hMSC differentiation into osteogenesis and adipogenesis in osteoporosis remain unclear. These considerations indicate that it is necessary to elucidate the relationship between adipogenic and osteogenic differentiation and to develop new drugs to prevent the differentiation of hMSCs into fat cells.

Bone morphogenetic proteins (BMPs) are multifunctional growth factors that belong to the transforming growth factor-beta (TGF-beta) superfamily (Halloran et al., 2020), and they have dual roles. The microenvironment is conducive to adipogenic or osteogenic differentiation, promoting either adipogenesis or osteogenesis (Atashi et al., 2015). TGF-beta is an important factor during bone formation and remodeling, and studies have indicated that TGF-beta could stimulate early differentiation of osteoblasts, while inhibiting late differentiation of osteoblasts into osteocytes (Tang and Alliston, 2013). The aims of the present study were to reveal the potential mechanisms underlying osteogenic and adipogenic differentiation of hMSCs and to investigate new targets for use in osteoporosis treatment. The microarray dataset GSE84500 from the Gene Expression Omnibus (GEO) database was used. Two groups were selected to identify differentially expressed genes (DEGs) related to osteogenic and adipogenic differentiation in hMSCs: a BMP2+3-isobutyl-1-methylxanthine (IBMX) group and a BMP2+IBMX+TGF-beta group. HMSCs were cultured under the same adipogenic conditions induced by BMP2 and IBMX, causing some to differentiate into adipocytes and others to differentiate into osteoblasts, and then TGF-beta was added to the culture. Functional and pathway enrichment analyses of DEGs were performed, and a protein–protein interaction (PPI) network was constructed to identify hub genes, which were verified at the mRNA expression level. MiRTarBase, TargetScan, and CyTargetLinker software were used to identify microRNAs that potentially regulate hub genes, providing a basis for further studies. The results of the current study may provide insight into the mechanisms of osteogenesis and adipogenesis and facilitate new therapeutic strategies for osteoporosis or obesity.

## Materials and Methods

### Microarray Data

The GEO dataset module from GEO database was selected (https://www.ncbi.nlm.nih.gov/geo/). An advanced search was then conducted as follows: ((osteoporosis) AND Bone marrow mesenchymal stem cells) AND “Expression profiling by array” [Filter]). The main purpose of this study was related to TGF-beta-induced osteogenic and adipogenic differentiation in hMSCs, and the inclusion organism of the dataset was *Homo sapiens*. Accordingly, only the mRNA microarray dataset GSE84500, which contains sufficient samples and four time-points, was available from the GEO database. The dataset includes normal hMSC samples from three different donors (van Zoelen et al., 2016). To better evaluate the TGF-beta-induced switch from adipogenic to osteogenic differentiation, 24 samples of hMSCs were selected from a BMP2+IBMX (BI) group and a BMP2+IBMX+TGF-beta (BIT) group. The two groups included 12 samples from 1, 2, 3, and 7 days of cell culture, with six samples at each time-point. This dataset platform was GPL570 ([HG-U133_Plus_2] Affymetrix Human Genome U133 Plus 2.0 Array).

### Identification of Differentially Expressed Genes

The GEO2R function (https://www.ncbi.nlm.nih.gov/geo/geo2r/) from the GEO database was used to identify DEGs in the BI and BIT groups. The original gene expression data were log2 converted, and DEG analysis was conducted with the default setting in GEO2R. DEGs with adjusted *p*-values <0.05 were considered statistically significant, and logFC ≥ 1 or logFC ≤ −1 was selected as the DEG threshold. Samples at each time-point were analyzed for upregulated and downregulated genes. In order to reduce false-positive results caused by operational error or culture conditions during cell experiments and to acquire stable genes, the intersections of the upregulated and downregulated genes of four time-points were used. Lastly, TGF-beta-mediated upregulated and downregulated genes were identified. A relative log expression (RLE) diagram was used to evaluate the quality of the sample chip, and a heatmap and a volcano plot were constructed using the pheatmap and gplots packages in R language, respectively.

### Gene Ontology and Kyoto Encyclopedia of Genes and Genomes Functional Analysis of Differentially Expressed Genes

To analyze the functions and potential pathways of the DEGs identified, the online DAVID software (https://david.ncifcrf.gov/) was used to perform Gene Ontology (GO) functional analysis (biological process = BP, cellular component = CC, and molecular function = MF) and Kyoto Encyclopedia of Genes and Genomes (KEGG) analysis. The Functional Annotation module was selected from the DAVID database, and the upregulated and downregulated genes were imported into the gene list of the Functional Annotation Tool for GO and KEGG analysis, respectively. The identifier selected wasofficial_gene_symbol, and the species selected was *H. sapiens*. Gene_ontology (BP/CC/MF) and pathways (kegg_pathway) were selected for enrichment analysis. Enrichment options were chosen from the default setting from the functional annotation chart, and *p*-value <0.05 was defined as statistically significant. According to the enrichment options, the eligible terms were screened out. Based on the *p*-value, the top six terms of GO functional enrichment terms (BP/CC/MF) were visualized with bar charts, and the ordinate is represented by–log10 (*p*-value).

### Protein–Protein Interaction Networks of Differentially Expressed Genes and Hub Gene Identification

The STRING database is an online tool designed to identify PPIs between DEGs from experiments and predictions (https://www.string-db.org/), and it was used to construct the PPI networks in the current study. All upregulated and downregulated genes were imported into the gene list. The criterion was medium confidence for selection ≥0.4, and *H. sapiens* was the selected organism*.* PPI networks were downloaded and deposited into Cytoscape v3.7.2 (https://cytoscape.org/), which was used to map interactions among the DEGs. The cytoHubba plugin from Cytoscape was then used to screen the hub genes of the PPI networks. To enhance data reliability, hub genes of upregulated and downregulated genes were obtained from the degree of intersection between MCC, MNC, and Degree modules.

### Construction of MiRNA–mRNA Interaction Networks

The CyTargetLinker4.1 plugin from Cytoscape (https://apps.cytoscape.org/apps/cytargetlinker) was used to predict miRNA–mRNA interaction networks. The Linksets module of the CyTargetLinker tutorial presentation (https://cytargetlinker.github.io/pages/tutorials/tutorial1) was used, and then the Linksets of MiRTarBase release 8.0 and TargetScan release 7.2 were selected (https://cytargetlinker.github.io/pages/linksets). Of these, the miRTarBase release 8.0 database is dedicated to collecting microRNA–target interactions (MTIs) with experimental evidence. Mirtarbase_hsa_8.0.xgmml.zip (https://cytargetlinker.github.io/pages/linksets/mirtarbase) included 502,652 MTIs, 15,038 target genes, and 2,595 microRNAs; and it was downloaded. Additionally, targetscan_hsa_7.2.xgmml.zip (https://cytargetlinker.github.io/pages/linksets/targetscan) included 264,563 MTIs, 13,077 target genes, and 405 microRNAs; and it was also downloaded. The first step of generating a miRNA–mRNA interaction network was the creation of txt files including upregulated genes and downregulated genes. The second step was the selection “File” from Cytoscape software → then “Network from File” → then “Import txt file.” The third step was importation of mirtarbase_hsa_8.0.xgmml and targetscan_hsa_7.2.xgmml into the CyTargetLinker component of Cytoscape software. The miRNA–mRNA interaction networks were thus constructed. An intersection threshold of 2 was set for the miRTarBase and TargetScan databases, and miRNA–mRNA interaction networks (hub miRNAs) were obtained and shared by the two databases.

### mRNA Expression Levels of Hub Genes and Validation

To investigate hub genes in the BI group and the BIT group, the mRNA expression levels of the top seven hub genes of upregulated and downregulated genes were identified from GSE84500. Each sample mRNA expression level for each time-point was obtained in two groups through R language and the GPL570 platform. The mRNA expression levels of 24 samples from four time-points were then combined, and they were divided into two groups of 12 samples. Lastly, the top seven hub genes in the BI group and BIT group were compared. The unpaired t-test was used for statistical analysis, and parameter testing and normality testing were conducted before the t-test. *p <* 0.05 was defined as a statistically significant difference. Statistical data are presented as the mean ± SD. GraphPad Prism (version 7.0) was used to conduct all statistical analyses and to generate graphs.

## Results

### Identification of Differentially Expressed Genes

**FIGURE 1:**
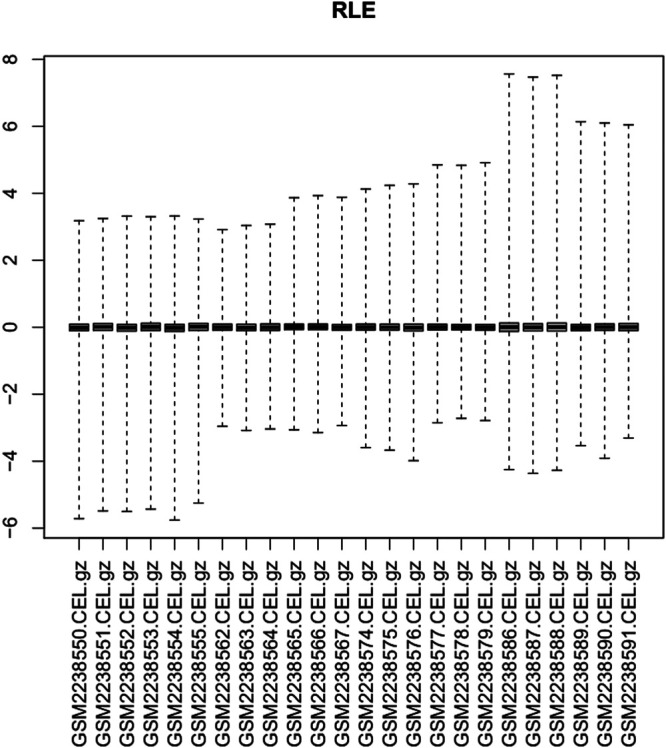
Relative log expression diagram of the 24 samples.

**FIGURE 2:**
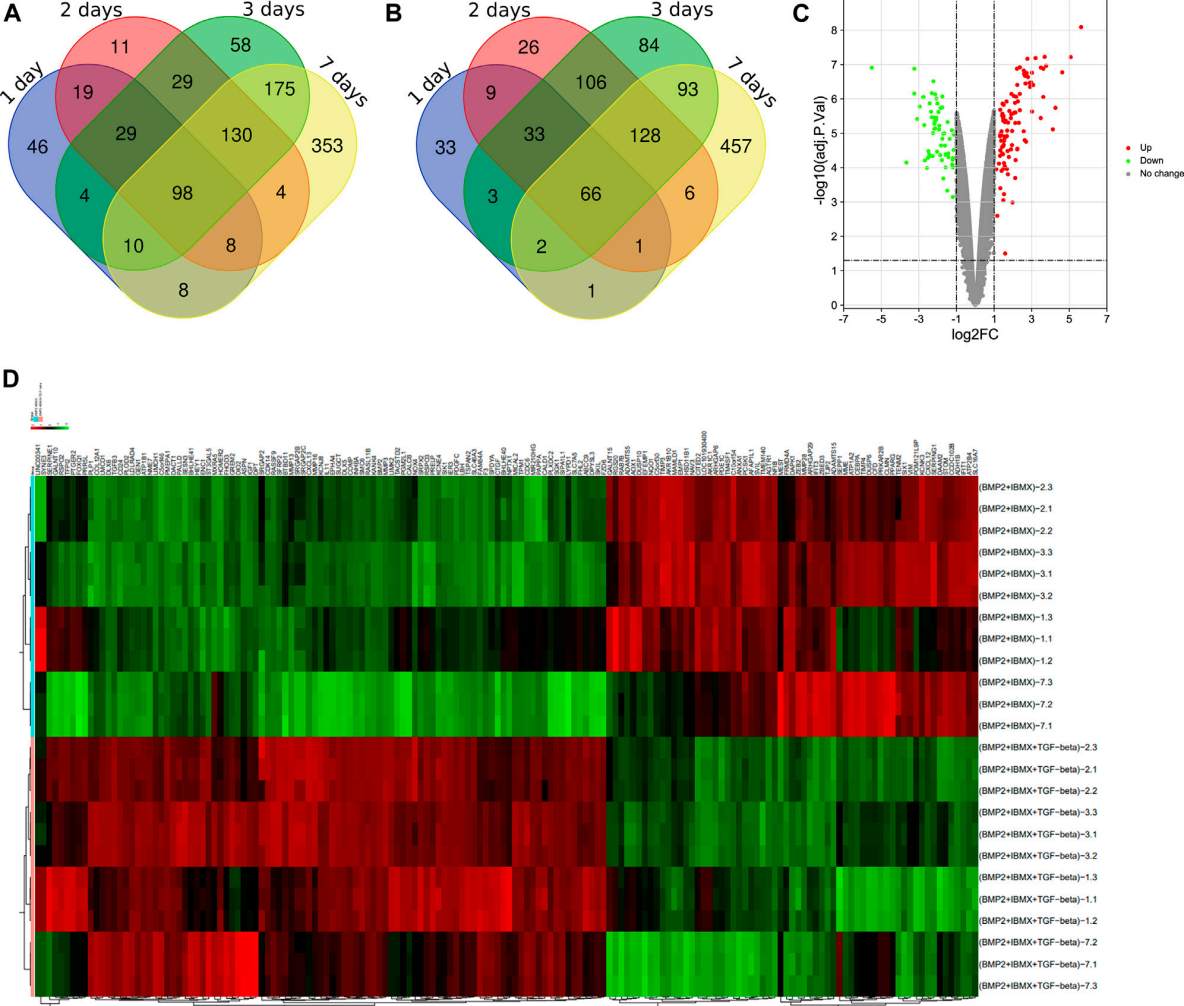
Venn diagrams showing **(A)** the 98 upregulated genes and **(B)** the 66 downregulated genes. A differentially expressed gene (DEG) volcano plot **(C)** and a heatmap **(D)** are shown. Red represents upregulated genes, and green represents downregulated genes (*p* < 0.05, logFC ≥ 1 or logFC ≤ −1).

Via filtering by set conditions, a total of 24 hMSC samples were acquired (Table 1). In evaluation of the quality of the sample chip, the median of 24 samples was almost on the same line and close to 0 (Figure 1), indicating superior quality of standardization. At the 1-day time-point, in the BIT group, 222 genes were upregulated in comparison with the BI group, in which 148 genes were downregulated. At the 2-day time-point, in the BIT group, 328 genes were upregulated in comparison with the BI group, in which 375 genes were downregulated. At the 3-day time-point, the corresponding numbers were 533 upregulated and 515 downregulated, and at the 7-day time-point, the corresponding numbers were 786 upregulated and 754 downregulated. The DEGs from the four time-points were combined, and the overlap of the final 98 upregulated and 66 downregulated genes was visualized as a Venn diagram (Figures 2A,B) and a volcano map (Figure 2C). Meanwhile, a heatmap for 164 DEGs from the log2 mRNA expression level of this microarray is shown (Figure 2D).

**TABLE 1:** Summary of the 24 samples.

Data number	Sample name
GSM2238550	hMSC, treated with BMP2+IBMX, 1-day differentiation
GSM2238551	hMSC, treated with BMP2+IBMX, 1-day differentiation
GSM2238552	hMSC, treated with BMP2+IBMX, 1-day differentiation
GSM2238553	hMSC, treated with BMP2+IBMX+TGFB, 1-day differentiation
GSM2238554	hMSC, treated with BMP2+IBMX+TGFB, 1-day differentiation
GSM2238555	hMSC, treated with BMP2+IBMX+TGFB, 1-day differentiation
GSM2238562	hMSC, treated with BMP2+IBMX, 2-day differentiation
GSM2238563	hMSC, treated with BMP2+IBMX, 2-day differentiation
GSM2238564	hMSC, treated with BMP2+IBMX, 2-day differentiation
GSM2238565	hMSC, treated with BMP2+IBMX+TGFB, 2-day differentiation
GSM2238566	hMSC, treated with BMP2+IBMX+TGFB, 2-day differentiation
GSM2238567	hMSC, treated with BMP2+IBMX+TGFB, 2-day differentiation
GSM2238574	hMSC, treated with BMP2+IBMX, 3-day differentiation
GSM2238575	hMSC, treated with BMP2+IBMX, 3-day differentiation
GSM2238576	hMSC, treated with BMP2+IBMX, 3-day differentiation
GSM2238577	hMSC, treated with BMP2+IBMX+TGFB, 3-day differentiation
GSM2238578	hMSC, treated with BMP2+IBMX+TGFB, 3-day differentiation
GSM2238579	hMSC, treated with BMP2+IBMX+TGFB, 3-day differentiation
GSM2238586	hMSC, treated with BMP2+IBMX, 7-day differentiation
GSM2238587	hMSC, treated with BMP2+IBMX, 7-day differentiation
GSM2238588	hMSC, treated with BMP2+IBMX, 7-day differentiation
GSM2238589	hMSC, treated with BMP2+IBMX+TGFB, 7-day differentiation
GSM2238590	hMSC, treated with BMP2+IBMX+TGFB, 7-day differentiation
GSM2238591	hMSC, treated with BMP2+IBMX+TGFB, 7-day differentiation

Note. hMSC, human bone marrow mesenchymal stem cell.

### Gene Ontology and Kyoto Encyclopedia of Genes and Genomes Functional Analysis of Differentially Expressed Genes

**FIGURE 3:**
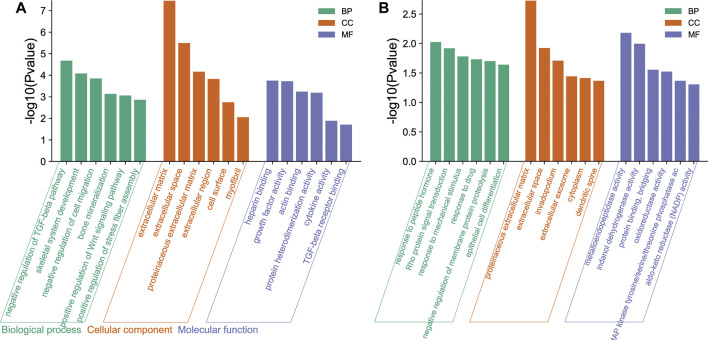
Gene Ontology (GO) functional enrichment of differentially expressed genes (DEGs) in osteogenic and adipogenic differentiation of human bone marrow mesenchymal stem cells (hMSCs). These genes were enriched in various biological process (BP), cellular component (CC), and molecular function (MF) terms. The ordinate is indicated on a −log10 (*p*-value) scale. **(A)** The main enrichment results of the upregulated genes. **(B)** The main enrichment results of the downregulated genes.

In GO functional analysis, upregulated and downregulated genes were enriched in various BP, CC, and MF terms (Figures 3A,B). In the BP category, the upregulated genes were mainly involved in the negative regulation of TGF-beta receptor pathway, skeletal system development, negative regulation of cell migration, and bone mineralization; the downregulated genes were mainly involved in the response to peptide hormone, Rho protein signal transduction, and response to mechanical stimulus. In the CC categories, the upregulated genes were mainly involved in extracellular matrix (ECM), extracellular space, proteinaceous ECM, and extracellular region; the downregulated genes were mainly involved in proteinaceous ECM, extracellular space, and invadopodium. Analysis of the MF category further demonstrated that the upregulated genes were mainly involved in heparin binding, growth factor activity, actin binding, and protein heterodimerization activity; the downregulated genes were mainly involved in metalloendopeptidase activity, indanol dehydrogenase activity, and protein binding bridging.

Five KEGG signaling pathways were identified (Tables 2, 3). The upregulated genes were primarily involved in three pathways, and the downregulated genes were primarily involved in two pathways. Although the *p*-value of “sa05200: Pathways in cancer” was >0.05, it contained a large number of enriched genes.

**TABLE 2:** KEGG pathways enrichment analyses of upregulated DEGs.

Category	Term	Count	*p*-Value	Genes
KEGG_PATHWAY	hsa04550: Signaling pathways regulating pluripotency of stem cells	6	0.0021	BMP2, DLX5, FZD6, IGF1, INHBA, SKIL
KEGG_PATHWAY	hsa04390: Hippo signaling pathway	5	0.0166	BMP2, TGFB3, FZD6, SERPINE1, CTGF
KEGG_PATHWAY	hsa04960: Aldosterone-regulated sodium reabsorption	3	0.0266	IGF1, ATP1B1, SGK1

Note. The three KEGG pathways were selected based on *p*-values.

KEGG, Kyoto Encyclopedia of Genes and Genomes; DEGs, differentially expressed genes.

**TABLE 3:** KEGG pathways enrichment analyses of downregulated DEGs.

Category	Term	Count	*p*-Value	Genes
KEGG_PATHWAY	hsa00980: Metabolism of xenobiotics by cytochrome P450	3	0.0384	HSD11B1, ADH1B, AKR1C1
KEGG_PATHWAY	hsa05200: Pathways in cancer	5	0.0806	CEBPA, CXCL12, DAPK1, AGTR1, PPARG

Note. The two KEGG pathways were selected based on *p*-values. Although the *p*-value of the “sa05200: Pathways in cancer” was >0.05, it contained a large number of enriched genes.

KEGG, Kyoto Encyclopedia of Genes and Genomes; DEGs, differentially expressed genes.

### Protein–Protein Interaction Networks of the Differentially Expressed Genes and Identification of Hub Genes

**FIGURE 4:**
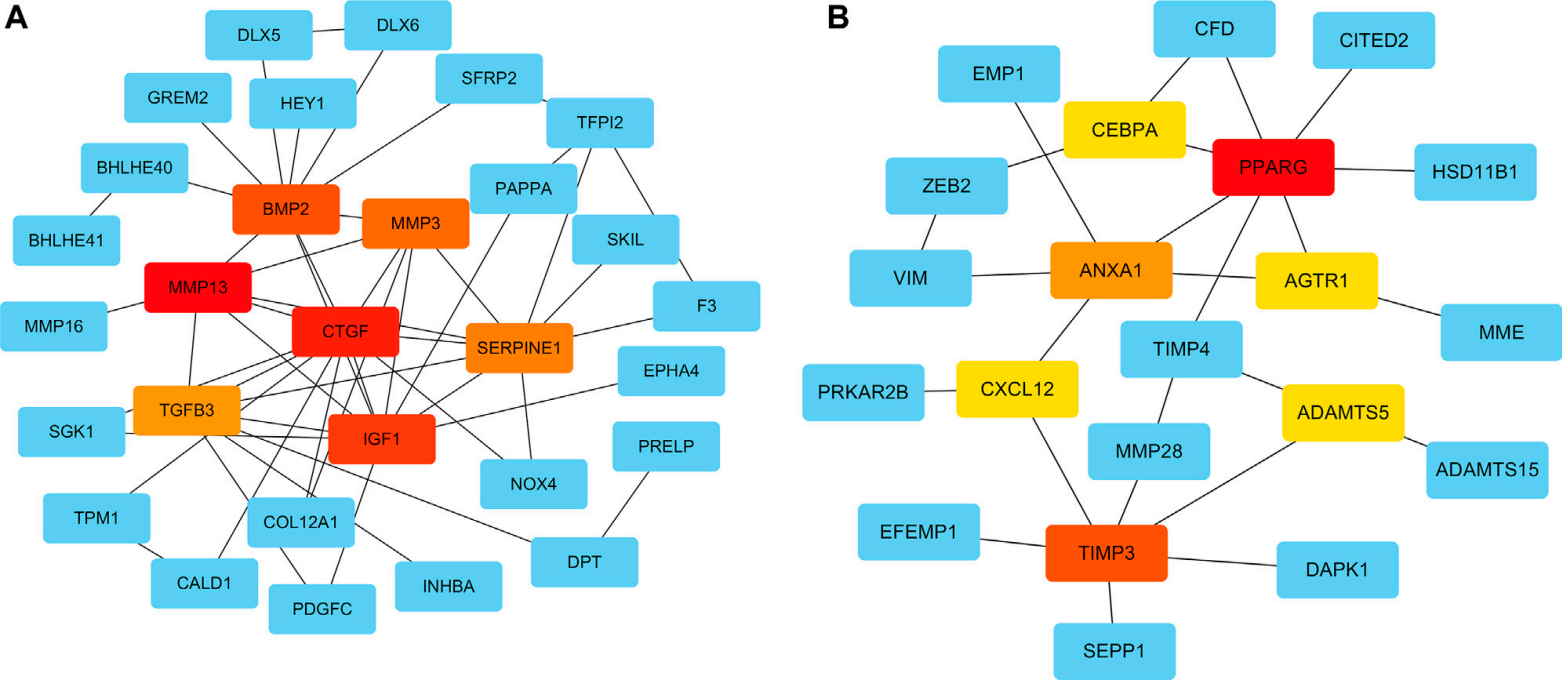
Protein–protein interaction (PPI) networks of the upregulated and downregulated genes were constructed using Cytoscape software. The top seven upregulated **(A)** and downregulated **(B)** hub genes, according to the MCC, MNC, and Degree modules of the cytoHubba were identified.

To systematically analyze the PPIs of DEGs, PPI networks of the upregulated and downregulated genes were constructed using Cytoscape software (Figures 4A,B). In the PPI networks of the upregulated genes, the DEGs with the highest connectivity degrees were BMP2, CTGF, IGF1, TGFB3, MMP13, MMP3, SERPINE1, COMP, ASPN, and IL11. Similarly, in the PPI networks of upregulated genes, the DEGs with the highest connectivity degrees were PPARG, TIMP3, ANXA1, ADAMTS5, TIMP4, AGTR1, NQO1, CXCL12, CEBPA, and CFD. The PPI networks of the DEGs from the STRING database were deposited into Cytoscape v3.7.2, and then the cytoHubba plugin from Cytoscape was used to identify hub genes of the PPI networks, and hub genes overlapped by MCC, MNC, and Degree. The top seven upregulated hub genes were CTGF, IGF1, BMP2, MMP13, TGFB3, MMP3, and SERPINE1; and the top seven downregulated hub genes were PPARG, TIMP3, ANXA1, ADAMTS5, AGTR1, CXCL12, and CEBPA (Figures 4A,B).

### Hub Gene mRNA Expression Levels and Validation

**FIGURE 5:**
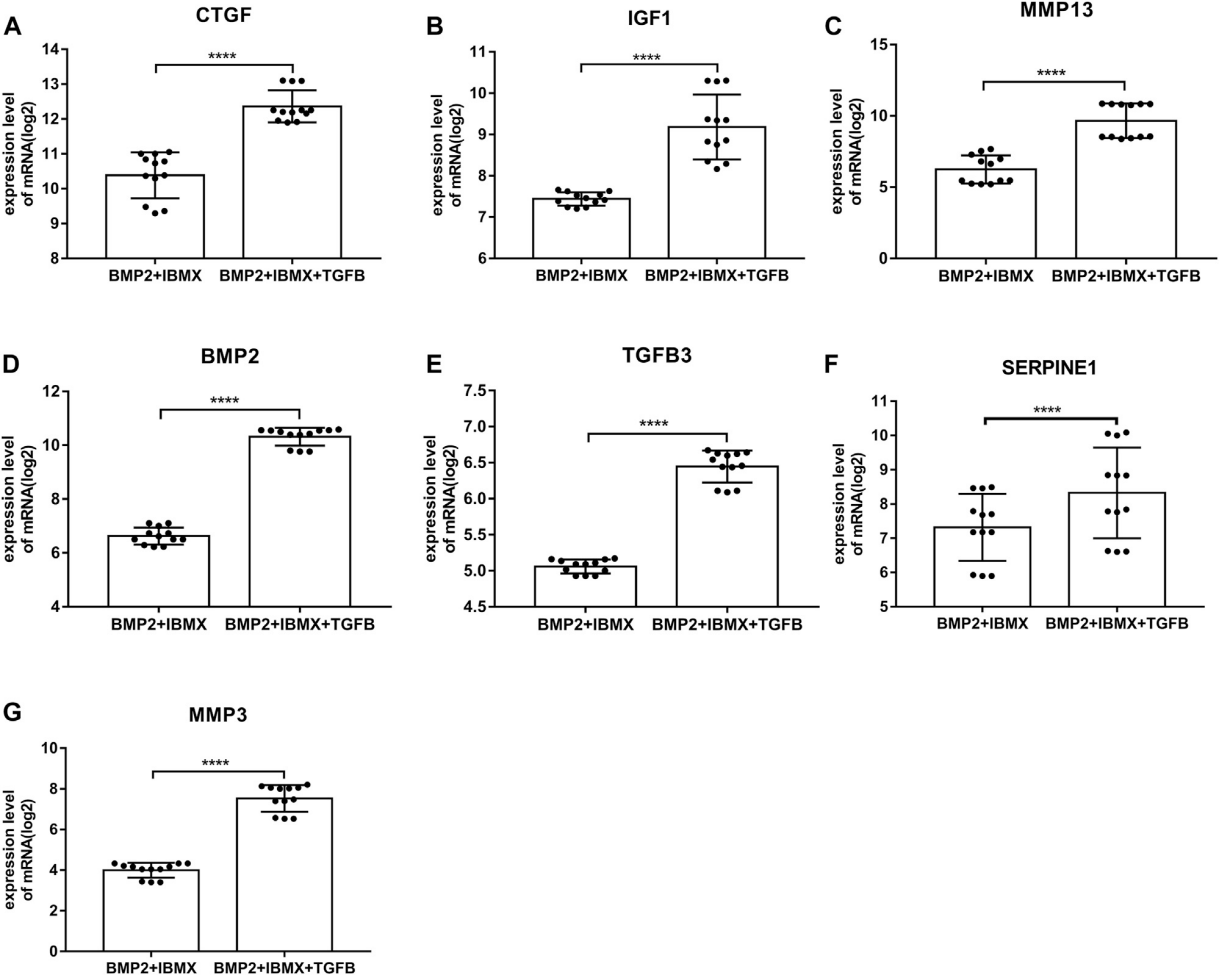
mRNA expression levels of the top seven upregulated hub genes involved in osteogenic differentiation, derived from analysis of 24 samples from four time-points (1, 2, 3, and 7 days; presented on a log2 scale). The data shown are means ± SD. *p < 0.05, **p < 0.01, ***p < 0.001, ****p < 0.0001.

**FIGURE 6:**
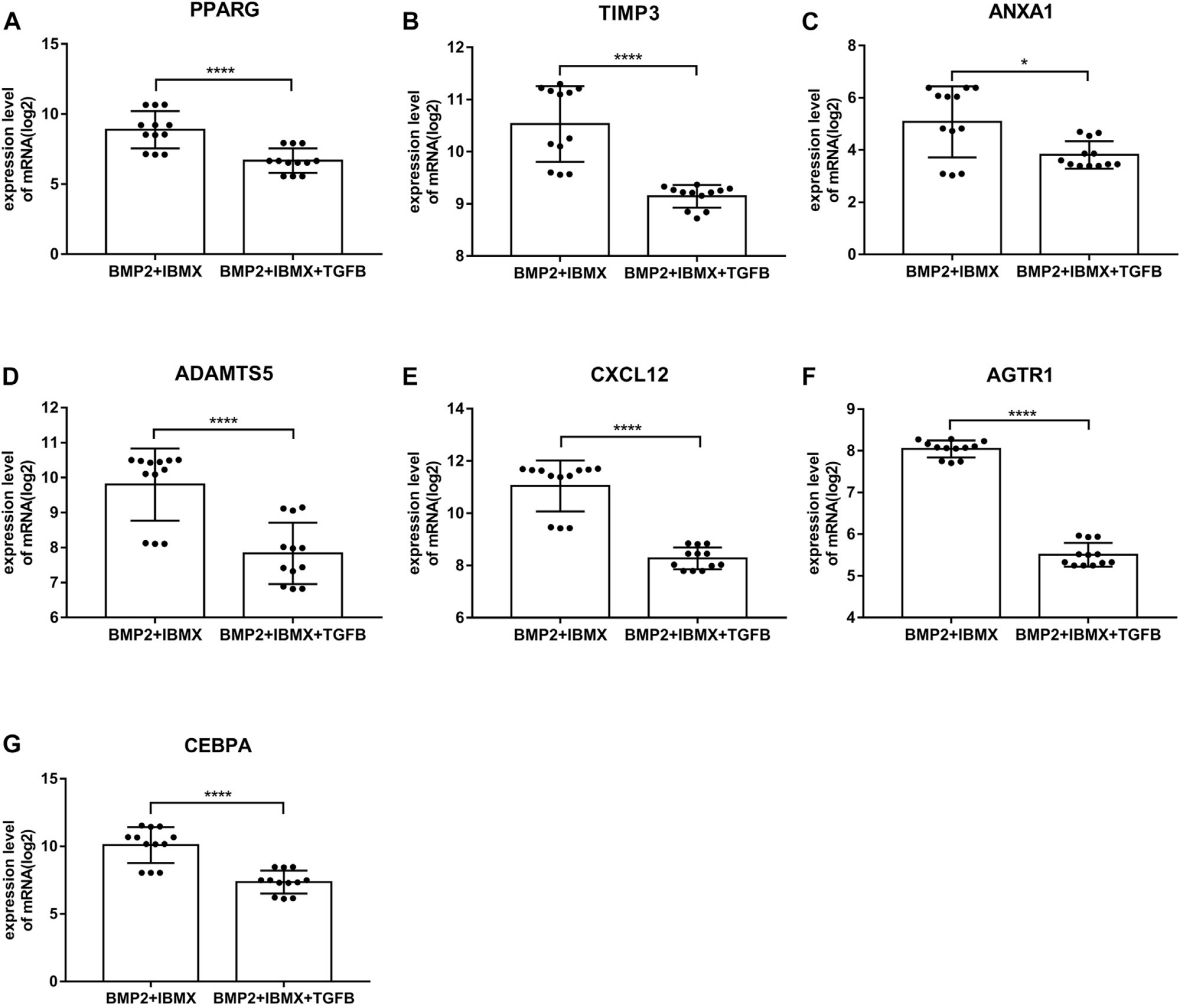
mRNA expression levels of the top seven downregulated hub genes involved in adipogenic differentiation, derived from analysis of 24 samples from four time-points (1, 2, 3, and 7 days; presented on a log2 scale). The data shown are means ± SD. *p < 0.05, **p < 0.01, ***p < 0.001, ****p < 0.0001.

mRNA expression levels of upregulated hub genes involved in osteogenic differentiation were significantly higher in the BIT group than in the BI group. However, the mRNA expression levels of downregulated hub genes involved in adipogenic differentiation were significantly lower in the BIT group than in the BI group. In statistical analyses, mRNA expression levels of all upregulated and downregulated hub genes differed significantly (Figures 5, 6). This indicated that the data were reliable and that these genes were hub genes for TGF-beta-induced upregulated and downregulated genes. These genes can be considered potential targets for TGF-beta-induced osteogenic and adipogenic differentiation of hMSCs.

### Construction of MiRNA–mRNA Interaction Networks

**FIGURE 7:**
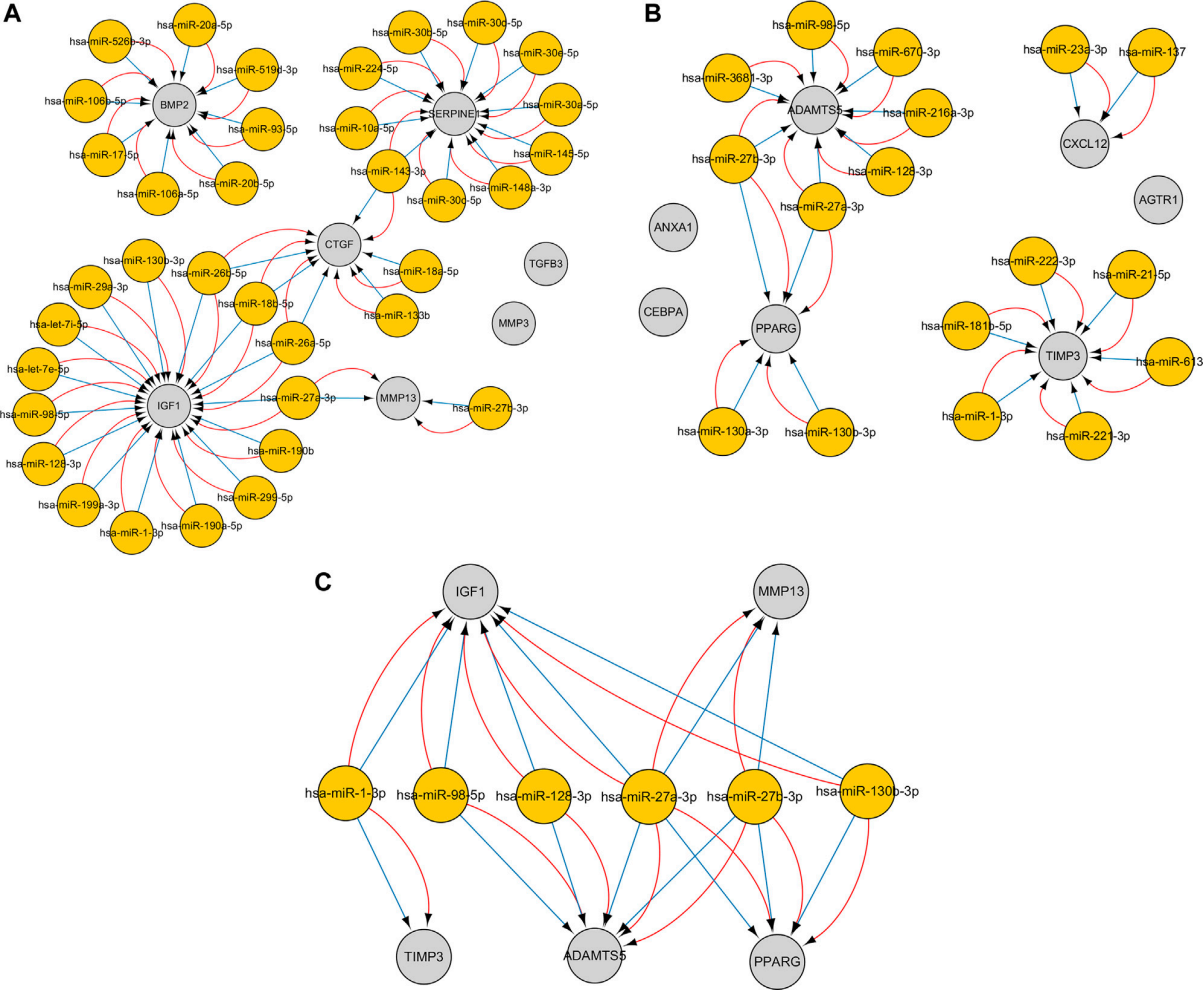
The miRNA–gene interaction networks for the top seven hub genes of the upregulated and downregulated genes were constructed. An overlap threshold was used to display only microRNA–target interactions (MTIs) in two regulatory interaction networks. Genes are indicated by gray circles, and miRNAs are indicated by yellow circles. Blue edges represent MTIs from the TargetScan v7.2 database, and red edges represent MTIs from the MiRTarBase v8.0 database. **(A)** MiRNA–gene pairs of the top seven hub genes of the upregulated genes. **(B)** MiRNA–gene pairs of the top seven hub genes of the downregulated genes. **(C)** Six miRNAs (hub miRNAs) coregulate two osteogenic genes and three adipogenic genes.

The CyTargetLinker plugin from Cytoscape was used to construct miRNA–gene interaction networks for the hub genes of the upregulated and downregulated genes. With respect to upregulated genes, 178 miRNAs were identified using the miRTarBase database, and 178 miRNAs were identified using the TargetScan database. With respect to downregulated genes, 93 miRNAs were identified using the miRTarBase database, and 150 miRNAs were identified using the TargetScan database. After setting an overlap threshold of two for the miRTarBase and TargetScan databases, 36 miRNAs were identified in the upregulated genes, and 17 miRNAs were identified in the downregulated genes. The miRNAs–genes are shown in Figures 7A–C. Specifically, 15 miRNAs that coregulate insulin growth factor 1 (IGF1), 10 miRNAs that coregulate SERPINE1, eight miRNAs that coregulate BMP2, six miRNAs that coregulate connective tissue growth factor (CTGF), two miRNAs that coregulate MMP13, seven miRNAs that coregulate ADAMTS5, six miRNAs that coregulate TIMP3, four miRNAs that coregulate PPARG, and two miRNAs that coregulate CXCL12 were identified; six miRNAs (hub miRNAs) that coregulate osteogenic genes and adipogenic genes were also identified (Table 4).

**TABLE 4:** Six hub miRNAs from the CyTargetLinker that coregulate five hub genes involved in osteogenic and adipogenic differentiation.

MiRNAs	The upregulated genes	The downregulated genes
hsa-miR-27a-3p	IGF1, MMP13	ADAMTS5, PPARG
hsa-miR-27b-3p	MMP13	ADAMTS5, PPARG
hsa-miR-128-3p	IGF1	ADAMTS5
hsa-miR-1-3p	IGF1	TIMP3
hsa-miR-98-5p	IGF1	ADAMTS5
hsa-miR-130b-3p	IGF1	PPARG

## Discussion

hMSCs are self-renewing precursor cells that can differentiate into bone, fat, cartilage, and stromal cells of the bone marrow (Frenette et al., 2013). It has been reported that they are ideal seed cells for bone tissue engineering (Fan et al., 2020). Notably, however, the effective cultivation of BMSCs requires a good culture environment and a good *in vitro* culture technique. With increased cell culture time, cell proliferation and stability may be reduced. In the GSE84500 dataset (van Zoelen et al., 2016), adipogenic differentiation of hMSCs increased within 3 days in an optimized medium. Adipogenic differentiation and proliferation entered a plateau phase or began to increase more slowly from 4 to 7 days. Therefore, cells cultured via the GSE84500 dataset are stable and available within 1 week. In order to reduce false-positive results caused by operational error or culture conditions during the cell experiments and to acquire stable genes, the intersection of the DEGs of four time-points was used in the present study. Differential expression was detected at all four time-points (1, 2, 3, and 7 days). This could reduce false-positive results caused by mistakes at a singular time-point.

In the current study, samples were obtained from hMSCs from the mRNA microarray dataset GSE84500 in GEO, undergoing osteogenic and adipogenic differentiation. Through bioinformatics analysis, a total of 164 DEGs were identified, including 98 upregulated genes involved in osteogenic differentiation and 66 downregulated genes involved in adipogenic differentiation. GO enrichment analysis indicated that the upregulated genes were associated with negative regulation of the TGF-beta receptor pathway, skeletal system development, negative regulation of cell migration, bone mineralization, ECM, and extracellular space. Upregulated genes were closely related to bone formation, confirming that osteogenic differentiation of hMSCs could be induced in an optimized microenvironment. Interestingly, the upregulated genes were significantly related to the ECM, which provides a local structural and signaling environment that controls cell proliferation, differentiation, migration, and communication during development (Laczko and Csiszar, 2020). In a previous study, optimized ECM could induce stronger osteogenic effects in mesenchymal stem cells (Freeman et al., 2019). In another recent study, it was reported that ECM mineralization was critical for osteogenesis, and its dysregulation could result in osteoporosis (Hao et al., 2020). The results of the current study are concordant with those previous results. The downregulated genes were involved in the response to peptide hormone, Rho protein signal transduction, responses to mechanical stimuli, proteinaceous ECM, and extracellular space. Peptide hormones such as adiponectin (Kim et al., 2016), parathyroid hormone (Ehrenmann et al., 2019), visfatin (Tsiklauri et al., 2018), and insulin can regulate the metabolism of human tissues and organs and are closely associated with lipid metabolism. Rho GTPases and Rho kinases regulate cell proliferation, migration, and apoptosis by influencing cytoskeletal dynamic stimulation and cell shape (Wang et al., 2017). It has also been shown that Rho GTPase signaling pathways are involved in the regulation of osteoclast activity (Morel et al., 2018). Rho and Rho-related kinase two are inactivated during adipogenesis, which enhances the expression of pro-adipogenic genes, and then induces actin stress fiber loss (Diep et al., 2018). The results of the present study are consistent with those previous reports. In the CC category of GO enrichment, proteinaceous ECM and extracellular space were the most enriched, indicating that intercellular signaling is essential for adipogenic differentiation. In enrichment of KEGG pathway analysis, the main enriched signaling pathways were those regulating pluripotency of stem cells and the Hippo signaling pathway. Studies suggest that the Hippo/YAP1 signaling pathway can promote osteogenic differentiation of mesenchymal stem cells and inhibit their adipogenic differentiation (Zhong et al., 2013; Pan et al., 2018). Li et al. (2021) also reported that the Hippo signaling pathway regulates exosomes from hMSCs to promote osteogenic differentiation and bone formation, preventing osteoporosis. There are currently few reports on the Hippo signaling cascade and TGF-beta in osteogenic differentiation; however, this warrants further research. The downregulated genes indicated that the main enriched component in KEGG analysis was metabolism of xenobiotics by cytochrome P450. In a recent study, repression of cytochrome P450 2b led to obesity (Heintz et al., 2019), and cytochrome P450 2E1 deficiency resulted in reduced adipogenesis (Dang and Yun, 2021).

On the basis of DEGs, PPI networks of upregulated and downregulated genes were created. The hub genes involved in osteogenic differentiation were CTGF, ICF1, BMP2, MMP13, TGFB3, MMP3, and SERPINE1. The hub genes involved in adipogenic differentiation were PPARG, TIMP3, ANXA1, ADAMTS5, AGTR1, and CXCL12. BMP2 was identified as a master regulator of the differentiation of osteoblasts (Scarfi, 2016), and its overexpression promoted osteogenesis in mesenchymal stems (Cai et al., 2021). Experimental research has suggested that BMP2 is the only growth factor capable of singly inducing bone formation (Cai et al., 2021). CTGF/CCN2 is a matricellular protein that is secreted into the ECM. It is considered a cell adhesion protein, and osteoblasts cultured on a CTGF matrix exhibited enhanced bone nodule formation and matrix mineralization (Hendesi et al., 2015). IGF1 is a multifunctional peptide growth factor that can induce strong proliferation and osteogenic differentiation in BMSCs (Wu et al., 2020b; Feng and Meng, 2021). During osteogenic differentiation, high expression of MMP13 in hMSCs grew on a type I collagen matrix. Additionally, knocking down MMP13 reduced the osteogenic differentiation of hMSCs on a type I collagen matrix (Arai et al., 2021). TGFB3 is a classic growth factor involved in bone generation (Yoon et al., 2018), and its overexpression upregulates alkaline phosphatase activity and induces the osteogenic differentiation of BMSCs (He et al., 2019). It also induces chondrogenesis of hMSCs (Uzieliene et al., 2021). Interactions between SERPINE1 and MMP3 and osteogenic differentiation have rarely been described, however, and warrant future research. Among the downregulated hub genes, peroxisome proliferator-activated receptor-gamma (PPARG) is a critical transcription factor of adipogenesis that is important in the formation of mature adipocytes (Stachecka et al., 2019). Some studies indicated that PPARG could be used as a new target for weight loss drugs. CEBPA acts as an adipogenic factor and is a key component in adipocyte differentiation (Gao et al., 2015). ADAMTS5 is the major protease that cleaves aggrecan; it reportedly promotes adipogenesis *in vitro* and *in vivo* in an established murine model (Bauters et al., 2016). PPARG, ADAMTS5, TIMP4, ANXA1, AGTR1, and CXCL12 genes are evidently associated with obesity, suggesting that the influence of these genes on obesity may be similar to the influence of fat accumulation in hMSCs. Furthermore, inhibitors of PPARG and ADAMTS5 can block the adipogenic differentiation of hMSCs (van Zoelen et al., 2016). Thus, these genes and corresponding inhibitors could be used as targets for drug development. To further confirm the accuracy of these hub genes, the mRNA expression levels of these hub genes were statistically analyzed. They were significantly higher in the BIT group than in the BI group, whereas the mRNA expression levels of the downregulated hub genes were significantly higher in the BI group than in the BIT group. This was because mesenchymal stem cells tend to differentiate into osteoblasts and inhibit adipogenic differentiation under the regulation of TGF-beta. All hub genes exhibited statistically significant differences. PPARG, ADAMTS5, AGTR1, and CXCL12 expression levels were consistent with a previous report (van Zoelen et al., 2016). Therefore, they are potential therapeutic targets for osteoporosis or obesity.

Integrated miRNA–mRNA regulatory networks of hub genes were constructed to improve understanding of potential molecular relationships between adipogenic differentiation and osteogenic differentiation in osteoporosis. To ensure the reliability and accuracy of the results, an overlap threshold of two was set for the miRTarBase and TargetScan databases to identify miRNA–gene interactions. Overall, 36 miRNAs were identified in the upregulated hub genes, which were mainly enriched in bone mineralization and the Hippo signaling pathway, whereas 17 miRNAs were identified in the downregulated hub genes, which were mainly enriched in the response to peptide hormone and pathways in cancer. Research has shown that a miRNA can target a number of genes, and a gene can be targeted by various miRNAs (Zhao et al., 2020). In the current study, a single gene was regulated by multiple miRNAs, and these miRNAs were experimentally validated. Interestingly, the results showed that some osteogenic genes and adipogenic genes were regulated by the same miRNA; for example, IGF1, MMP13, PPARG, and ADAMTS5 were regulated by hsa-miR-27a-3p; and ADAMTS5, PPARG, and MMP13 were regulated by hsa-miR-27b-3p. This may be because the miRNAs have the ability to bidirectionally regulate target genes. For example, a miR-149-3p mimic reduced the adipogenic differentiation potential of BMSCs and enhanced their osteogenic differentiation potential (Li et al., 2019). These hub miRNA–mRNA pairs may be therapeutic targets in osteoporosis.

In the current study, an integrated bioinformatics approach and strict screening conditions were used to process datasets. Hub genes were verified using the unpaired t-test. But the study had some limitations. The number of samples in the dataset was small, and larger samples are needed to confirm the study results. The study was based on microarray data obtained *in vitro*, and more *in vitro* and *in vivo* experiments are required to further verify the results. Lastly, the specific regulatory relationship between miRNAs and mRNAs was not further confirmed, and the transformation relationship between adipogenic differentiation and osteogenic differentiation required further confirmation. Nonetheless, we think that the results of the study are valuable and reliable. Identification of the DEGs was derived from the intersection of four time points, which reduced the likelihood of false-positive results. Most of the downregulated hub genes were consistent with van Zoelen et al. (2016). Hub miRNAs were selected from the intersection of two databases, of which miRTarBase is dedicated to collecting MTIs with experimental evidence. These results could provide a reference on osteoporosis or senile obesity, or for bioinformatics research, but more experiments are needed to support the results of the present study.

## Conclusion

Microarray and bioinformatics approaches were used to identify DEGs involved in adipogenic differentiation and osteogenic differentiation in hMSCs and to identify functions and pathways that the DEGs were involved in. Hub genes of osteogenic differentiation and adipogenic differentiation were identified, and their miRNA–mRNA regulation networks were constructed. The study provides new insight into the osteogenic differentiation and adipogenic differentiation of hMSCs. The hub genes/miRNAs identified may provide a basis for the screening of biomarkers related to osteoporosis or obesity, or for developing new therapies and drugs for osteoporosis or obesity.

## Data Availability

The datasets presented in this study can be found in online repositories. The names of the 360 repository/repositories and accession number(s) can be found in the article/Supplementary Material.

## References

[B1] Arai Y. Choi B. Kim B. J. Park S. Park H. Moon J. J. (2021). Cryptic Ligand on Collagen Matrix Unveiled by MMP13 Accelerates Bone Tissue Regeneration via MMP13/Integrin α3/RUNX2 Feedback Loop. Acta Biomater. 125, 219–230. 10.1016/j.actbio.2021.02.042 33677160

[B2] Atashi F. Modarressi A. Pepper M. S. (2015). The Role of Reactive Oxygen Species in Mesenchymal Stem Cell Adipogenic and Osteogenic Differentiation: A Review. Stem Cells Dev. 24 (10), 1150–1163. 10.1089/scd.2014.0484 25603196 PMC4424969

[B3] Bauters D. Scroyen I. Deprez-Poulain R. Lijnen H. R. (2016). ADAMTS5 Promotes Murine Adipogenesis and Visceral Adipose Tissue Expansion. Thromb. Haemost. 116 (4), 694–704. 10.1160/TH16-01-0015 27383908

[B4] Black D. M. Rosen C. J. (2016). Postmenopausal Osteoporosis. N. Engl. J. Med. 374 (3), 254–262. 10.1056/NEJMcp1513724 26789873

[B5] Cai H. Zou J. Wang W. Yang A. (2021). BMP2 Induces hMSC Osteogenesis and Matrix Remodeling. Mol. Med. Rep. 23 (2), 125. 10.3892/mmr.2020.11764 33300084 PMC7751477

[B6] Chang P. Y. Feldman D. Stefanick M. L. McDonnell D. P. Thompson B. M. McDonald J. G. (2019). 27‐Hydroxycholesterol, an Endogenous SERM, and Risk of Fracture in Postmenopausal Women: A Nested Case‐Cohort Study in the Women's Health Initiative. J. Bone Miner. Res. 34 (1), 59–66. 10.1002/jbmr.3576 30138538 PMC6478389

[B7] Dang T. T. H. Yun J. W. (2021). Cytochrome P450 2E1 (CYP2E1) Positively Regulates Lipid Catabolism and Induces browning in 3T3-L1 white Adipocytes. Life Sci. 278, 119648. 10.1016/j.lfs.2021.119648 34043994

[B8] Diep D. T. V. Hong K. Khun T. Zheng M. Ul-Haq A. Jun H.-S. (2018). Anti-adipogenic Effects of KD025 (SLx-2119), a ROCK2-specific Inhibitor, in 3T3-L1 Cells. Sci. Rep. 8 (1), 2477. 10.1038/s41598-018-20821-3 29410516 PMC5802830

[B9] Duque G. (2008). Bone and Fat Connection in Aging Bone. Curr. Opin. Rheumatol. 20 (4), 429–434. 10.1097/BOR.0b013e3283025e9c 18525356

[B10] Eastell R. Rosen C. J. Black D. M. Cheung A. M. Murad M. H. Shoback D. (2019). Pharmacological Management of Osteoporosis in Postmenopausal Women: An Endocrine Society* Clinical Practice Guideline. J. Clin. Endocrinol. Metab. 104 (5), 1595–1622. 10.1210/jc.2019-00221 30907953

[B11] Ehrenmann J. Schöppe J. Klenk C. Plückthun A. (2019). New Views into Class B GPCRs from the crystal Structure of PTH1R. FEBS J. 286 (24), 4852–4860. 10.1111/febs.15115 31670461

[B12] Ensrud K. E. Crandall C. J. (2017). Osteoporosis. Ann. Intern. Med. 167 (3), ITC17–C32. 10.7326/AITC201708010 28761958

[B13] Fan T. Qu R. Yu Q. Sun B. Jiang X. Yang Y. (2020). Bioinformatics Analysis of the Biological Changes Involved in the Osteogenic Differentiation of Human Mesenchymal Stem Cells. J. Cel. Mol. Med. 24 (14), 7968–7978. 10.1111/jcmm.15429 PMC7348183 32463168

[B14] Feng J. Meng Z. (2021). Insulin Growth Factor-1 P-romotes the P-roliferation and O-steogenic D-ifferentiation of B-one M-arrow M-esenchymal S-tem C-ells through the Wnt/β-catenin P-athway. Exp. Ther. Med. 22 (2), 891. 10.3892/etm.2021.10323 34194569 PMC8237273

[B15] Freeman F. Browe D. C. Browe D. Nulty J. Von Euw S. Grayson W. (2019). Biofabrication of Multiscale Bone Extracellular Matrix Scaffolds for Bone Tissue Engineering. eCM 38, 168–187. 10.22203/eCM.v038a12 31602629

[B16] Frenette P. S. Pinho S. Lucas D. Scheiermann C. (2013). Mesenchymal Stem Cell: Keystone of the Hematopoietic Stem Cell Niche and a Stepping-Stone for Regenerative Medicine. Annu. Rev. Immunol. 31, 285–316. 10.1146/annurev-immunol-032712-095919 23298209

[B17] Gao Y. Sun Y. Duan K. Shi H. Wang S. Li H. (2015). CpG Site DNA Methylation of theCCAAT/enhancer-Binding Protein, Alphapromoter in Chicken Lines Divergently Selected for Fatness. Anim. Genet. 46 (4), 410–417. 10.1111/age.12326 26156393

[B18] Haasters F. Docheva D. Gassner C. Popov C. Böcker W. Mutschler W. (2014). Mesenchymal Stem Cells from Osteoporotic Patients Reveal Reduced Migration and Invasion upon Stimulation with BMP-2 or BMP-7. Biochem. Biophysical Res. Commun. 452 (1), 118–123. 10.1016/j.bbrc.2014.08.055 25152406

[B19] Halloran D. Durbano H. W. Nohe A. (2020). Bone Morphogenetic Protein-2 in Development and Bone Homeostasis. Jdb 8 (3), 19. 10.3390/jdb8030019 PMC7557435 32933207

[B20] Hao Q. Liu Z. Lu L. Zhang L. Zuo L. (2020). Both JNK1 and JNK2 Are Indispensable for Sensitized Extracellular Matrix Mineralization in IKKβ-Deficient Osteoblasts. Front. Endocrinol. 11, 13. 10.3389/fendo.2020.00013 PMC7028708 32117051

[B21] He W. Chen L. Huang Y. Xu Z. Xu W. Ding N. (2019). Synergistic Effects of Recombinant Lentiviral-Mediated BMP2 and TGF-Beta3 on the Osteogenic Differentiation of Rat Bone Marrow Mesenchymal Stem Cells *In Vitro* . Cytokine 120, 1–8. 10.1016/j.cyto.2019.03.020 30991228

[B22] Heintz M. M. Kumar R. Rutledge M. M. Baldwin W. S. (2019). Cyp2b-null Male Mice Are Susceptible to Diet-Induced Obesity and Perturbations in Lipid Homeostasis. J. Nutr. Biochem. 70, 125–137. 10.1016/j.jnutbio.2019.05.004 31202118 PMC6642837

[B23] Hendesi H. Barbe M. F. Safadi F. F. Monroy M. A. Popoff S. N. (2015). Integrin Mediated Adhesion of Osteoblasts to Connective Tissue Growth Factor (CTGF/CCN2) Induces Cytoskeleton Reorganization and Cell differentiationJournal Article; Research Support. PLoS One 10 (2), e0115325. 10.1371/journal.pone.0115325 25714841 PMC4340870

[B24] Hu L. Yin C. Zhao F. Ali A. Ma J. Qian A. (2018). Mesenchymal Stem Cells: Cell Fate Decision to Osteoblast or Adipocyte and Application in Osteoporosis Treatment. Ijms 19 (2), 360. 10.3390/ijms19020360 PMC5855582 29370110

[B25] Kim H. Y. Bae E. H. Ma S. K. Chae D. W. Choi K. H. Kim Y.-S. (2016). Association of Serum Adiponectin Level with Albuminuria in Chronic Kidney Disease Patients. Clin. Exp. Nephrol. 20 (3), 443–449. 10.1007/s10157-015-1173-4 26445954

[B26] Laczko R. Csiszar K. (2020). Lysyl Oxidase (LOX): Functional Contributions to Signaling Pathways. Biomolecules 10 (8), 1093. 10.3390/biom10081093 PMC7465975 32708046

[B27] Li L. Zhou X. Zhang J.-t. Liu A.-f. Zhang C. Han J.-c. (2021). Exosomal miR-186 Derived from BMSCs Promote Osteogenesis through Hippo Signaling Pathway in Postmenopausal Osteoporosis. J. Orthop. Surg. Res. 16 (1), 23. 10.1186/s13018-020-02160-0 33413543 PMC7791800

[B28] Li Y. Yang F. Gao M. Gong R. Jin M. Liu T. (2019). MiR-149-3p Regulates the Switch between Adipogenic and Osteogenic Differentiation of BMSCs by Targeting FTO. Mol. Ther. - Nucleic Acids 17, 590–600. 10.1016/j.omtn.2019.06.023 31382190 PMC6690430

[B29] McClung M. R. O'Donoghue M. L. Papapoulos S. E. Bone H. Langdahl B. Saag K. G. (2019). Odanacatib for the Treatment of Postmenopausal Osteoporosis: Results of the LOFT Multicentre, Randomised, Double-Blind, Placebo-Controlled Trial and LOFT Extension Study. Lancet Diabetes Endocrinol. 7 (12), 899–911. 10.1016/S2213-8587(19)30346-8 31676222

[B30] Morel A. Blangy A. Vives V. (2018). Methods to Investigate the Role of Rho GTPases in Osteoclast Function. Methods Mol. Biol. 1821, 219–233. 10.1007/978-1-4939-8612-5_15 30062415

[B31] Pan J.-X. Xiong L. Zhao K. Zeng P. Wang B. Tang F.-L. (2018). YAP Promotes Osteogenesis and Suppresses Adipogenic Differentiation by Regulating β-catenin Signaling. Bone Res. 6, 18. 10.1038/s41413-018-0018-7 29872550 PMC5984632

[B32] Rosen C. J. Bouxsein M. L. (2006). Mechanisms of Disease: Is Osteoporosis the Obesity of Bone? Nat. Rev. Rheumatol. 2 (1), 35–43. 10.1038/ncprheum0070 16932650

[B33] Sambrook P. Cooper C. (2006). Osteoporosis. The Lancet 367 (9527), 2010–2018. 10.1016/S0140-6736(06)68891-0 16782492

[B34] Scarfì S. (2016). Use of Bone Morphogenetic Proteins in Mesenchymal Stem Cell Stimulation of Cartilage and Bone Repair. Wjsc 8 (1), 1–12. 10.4252/wjsc.v8.i1.1 26839636 PMC4723717

[B35] Souza A. T. P. Freitas G. P. Lopes H. B. Totoli G. G. C. Tarone A. G. Marostica-Junior M. R. (2021). Jabuticaba Peel Extract Modulates Adipocyte and Osteoblast Differentiation of MSCs from Healthy and Osteoporotic Rats. J. Bone Miner. Metab. 39 (2), 163–173. 10.1007/s00774-020-01152-8 32889573

[B36] Stachecka J. Nowacka-Woszuk J. Kolodziejski P. A. Szczerbal I. (2019). The Importance of the Nuclear Positioning of the PPARG Gene for its Expression during Porcine *In Vitro* Adipogenesis. Chromosome Res. 27 (3), 271–284. 10.1007/s10577-019-09604-2 30656515 PMC6733831

[B37] Tang S. Y. Alliston T. (2013). Regulation of Postnatal Bone Homeostasis by TGFβ. Bonekey Rep. 2, 255. 10.1038/bonekey.2012.255 24404376 PMC3722719

[B38] Tsiklauri L. Werner J. Kampschulte M. Frommer K. W. Berninger L. Irrgang M. (2018). Visfatin Alters the Cytokine and Matrix-Degrading Enzyme Profile during Osteogenic and Adipogenic MSC Differentiation. Osteoarthritis and Cartilage 26 (9), 1225–1235. 10.1016/j.joca.2018.06.001 29908226

[B39] Uzieliene I. Bagdonas E. Hoshi K. Sakamoto T. Hikita A. Tachtamisevaite Z. (2021). Different Phenotypes and Chondrogenic Responses of Human Menstrual Blood and Bone Marrow Mesenchymal Stem Cells to Activin A and TGF-Β3. Stem Cell Res. Ther. 12 (1), 251. 10.1186/s13287-021-02286-w 33926568 PMC8082646

[B40] van Zoelen E. J. Duarte I. Hendriks J. M. van der Woning S. P. (2016). Tgfβ-Induced Switch from Adipogenic to Osteogenic Differentiation of Human Mesenchymal Stem Cells: Identification of Drug Targets for Prevention of Fat Cell Differentiation. Stem Cell Res. Ther. 7 (1), 123. 10.1186/s13287-016-0375-3 27562730 PMC5000485

[B41] Vellucci R. Mediati R. D. Ballerini G. (2014). Use of Opioids for Treatment of Osteoporotic Pain. ccmbm 11 (3), 173–176. 10.11138/ccmbm/2014.11.3.173 PMC4269138 25568648

[B42] Wang T. Kang W. Du L. Ge S. (2017). Rho-kinase Inhibitor Y-27632 Facilitates the Proliferation, Migration and Pluripotency of Human Periodontal Ligament Stem Cells. J. Cell. Mol. Med. 21 (11), 3100–3112. 10.1111/jcmm.13222 28661039 PMC5661246

[B43] Wu J. Cai P. Lu Z. Zhang Z. He X. Zhu B. (2020a). Identification of Potential Specific Biomarkers and Key Signaling Pathways between Osteogenic and Adipogenic Differentiation of hBMSCs for Osteoporosis Therapy. J. Orthop. Surg. Res. 15 (1), 437. 10.1186/s13018-020-01965-3 32967719 PMC7510089

[B44] Wu L. Zhang G. Guo C. Pan Y. (2020b). Intracellular Ca2^+^ Signaling Mediates IGF-1-Induced Osteogenic Differentiation in Bone Marrow Mesenchymal Stem Cells. Biochem. Biophysical Res. Commun. 527 (1), 200–206. 10.1016/j.bbrc.2020.04.048 32446367

[B45] Yoon S.-J. Yoo Y. Nam S. Hyun H. Lee D.-W. Um S. (2018). The Cocktail Effect of BMP-2 and TGF-Β1 Loaded in Visible Light-Cured Glycol Chitosan Hydrogels for the Enhancement of Bone Formation in a Rat Tibial Defect Model. Mar. Drugs 16 (10), 351. 10.3390/md16100351 PMC6213427 30257482

[B46] Yu W. Zhong L. Yao L. Wei Y. Gui T. Li Z. (2021). Bone Marrow Adipogenic Lineage Precursors Promote Osteoclastogenesis in Bone Remodeling and Pathologic Bone Loss. J. Clin. Invest. 131 (2), e140214. 10.1172/JCI140214 PMC7810488 33206630

[B47] Zhao H. Chang A. Ling J. Zhou W. Ye H. Zhuo X. (2020). Construction and Analysis of miRNA-mRNA Regulatory Networks in the Radioresistance of Nasopharyngeal Carcinoma. 3 Biotech. 10 (12), 511. 10.1007/s13205-020-02504-x PMC7648832 33184596

[B48] Zhi F. Ding Y. Wang R. Yang Y. Luo K. Hua F. (2021). Exosomal Hsa_circ_0006859 Is a Potential Biomarker for Postmenopausal Osteoporosis and Enhances Adipogenic versus Osteogenic Differentiation in Human Bone Marrow Mesenchymal Stem Cells by Sponging miR-431-5p. Stem Cel Res. Ther. 12 (1), 157. 10.1186/s13287-021-02214-y PMC7923524 33648601

[B49] Zhong W. Tian K. Zheng X. Li L. Zhang W. Wang S. (2013). Mesenchymal Stem Cell and Chondrocyte Fates in a Multishear Microdevice Are Regulated by Yes-Associated Protein. Stem Cells Dev. 22 (14), 2083–2093. 10.1089/scd.2012.0685 23442010

