# Supplementary material for: TGF-Beta Induced Key Genes of Osteogenic and Adipogenic Differentiation in Human Mesenchymal Stem Cells and MiRNA–mRNA Regulatory Networks
Source: Front Genet. 2021 Nov 25;12:759596. doi: 10.3389/fgene.2021.759596 (PMC8656281; doi:10.3389/fgene.2021.759596)
Supplement: Supplementary file 1 [file DataSheet1.docx]

Supplementary Material

# Supplementary Figures and Tables

## Supplementary Figure 1


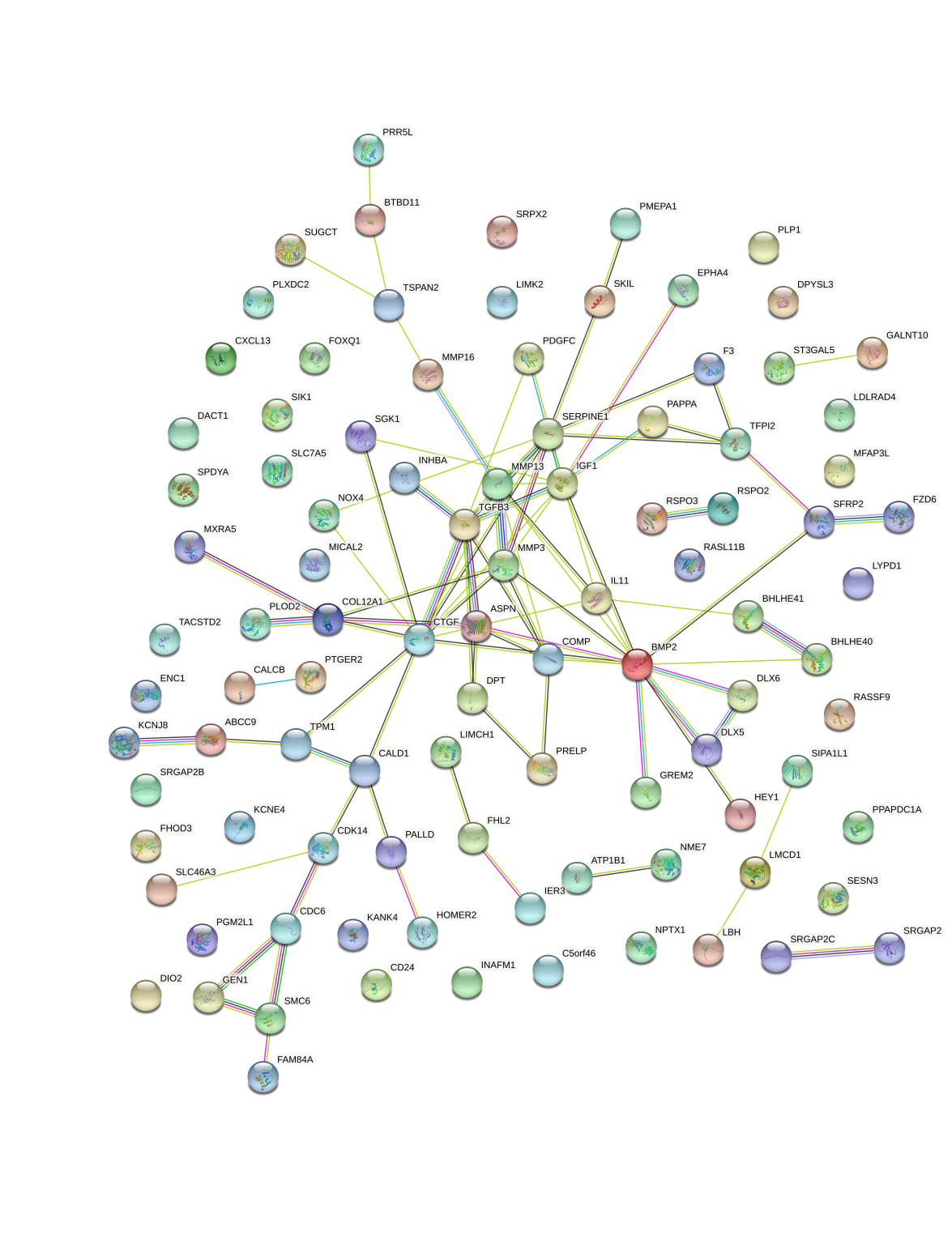


**Supplementary Figure 1.** PPI interaction networks of 98 downregulated genes based on STRING database

## Supplementary Figure 2


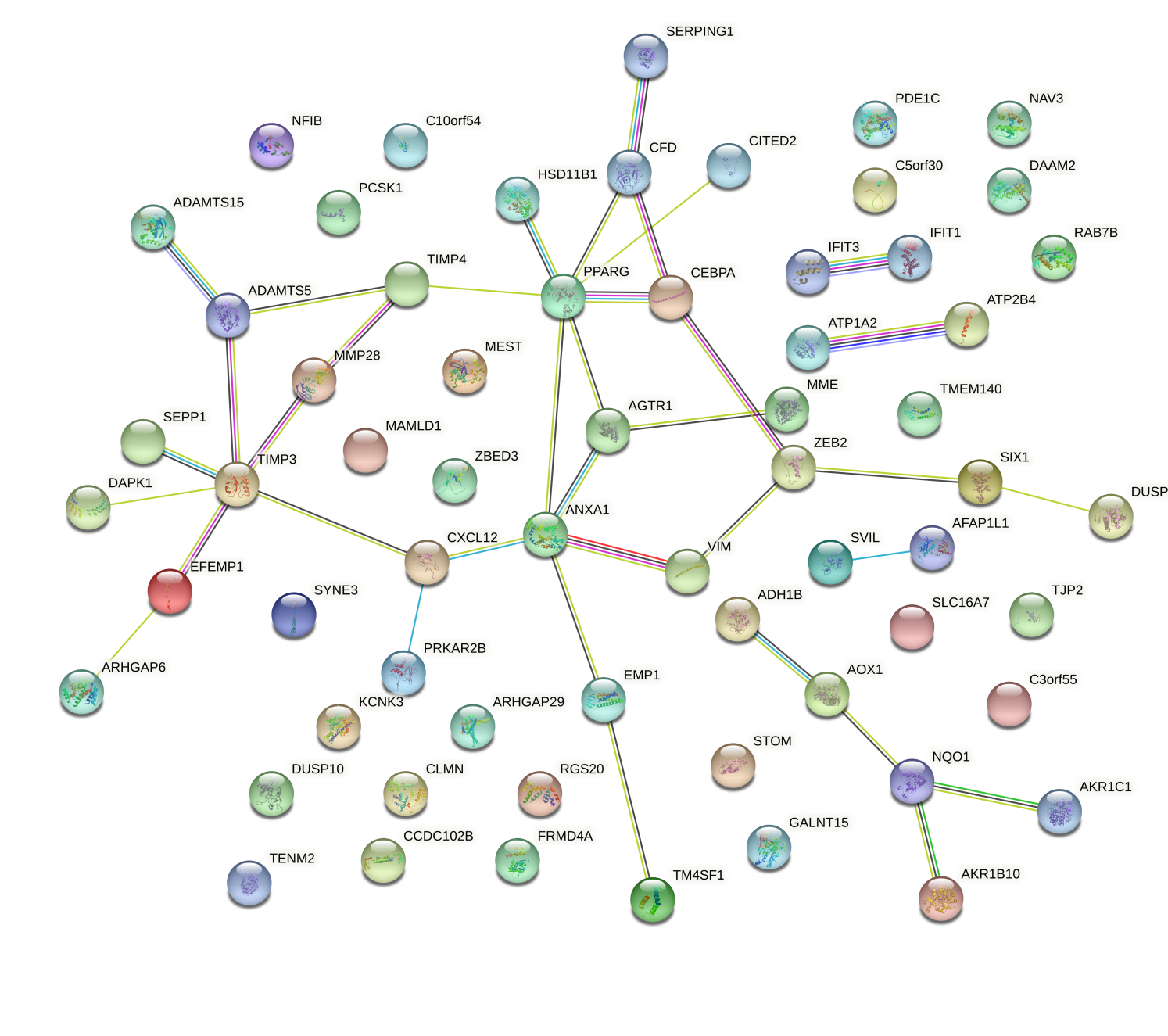


**Supplementary Figure 2.** PPI interaction networks of 66 downregulated genes based on STRING database

## Supplementary Table 1

| Category | genes |
| --- | --- |
| 98 upregulated genes | ABCC9、ASPNA、TP1B1、BHLHE40、BHLHE41、BMP2、BTBD11、C5orf46、CAL、CALD1、CD24、CDC6、CDK14、COL12A1、COMP、CTGF、CXCL13、DACT1、DIO、DLX5、DLX6、DPT、DPYSL3、ENC1、EPHA4、F3、FAM84A、FHL2、FHOD3、FOXQ1、FZD6、GALNT10、GEN1、GREM2、HEY1、HOMER2、IER3、IGF1、IL11、INAFM1、INHBA、KANK4、KCNE4、KCNJ8、LBH、LDLRAD4、LIMCH1、LIMK2、LMCD1、LYPD1、MFAP3L、MICAL2、MIR210HG、MMP13、MMP16、MMP3、MXRA5、NME7、NOX4、NPTX1、PALLD、PAPPA、PDGFC、PGM2L1、PLOD2、PLP1、PLPP4、PLXDC2、PMEPA1、PRELP、PRR5L、PTGER2、RASL11B、RASSF9、RSPO2、RSPO3、SERPINE1、SESN3、SFRP2、SGK1、SIK1、SIPA1L1、SKIL、SLC46A3、SLC7A5、SMC6、SPDYA、SRGAP2C、SRPX2、ST3GAL5、SUGCT、TACSTD2、TFPI2、TGFB3、TPM1、TSPAN2、SRGAP2B、SRGAP2 |
| 66 downregulated genes | ADAMTS15、ADAMTS5、ADH1B、AFAP1L1、AGTR1、AKR1B10、ANXA1、AOX1、ARHGAP29、ARHGAP6、ATP1A2、ATP2B4、C10orf54、C5orf30、CCDC102B、CEBPA、CFD、CITED2、CLMN、CXCL12、DAAM2、DAPK1、DUSP10、DUSP6、EFEMP1、EMP1、FRMD4A、GALNT15、HSD11B1、IFIT1、IFIT3、KCNK3、LOC101930400、MAMLD1、MEST、MME、MMP28、NAV3、NFIB、NQO1、PCSK1、PDE1C、POM121L9P、PPARG、PQLC2L、PRKAR2B、RAB7B、RGS20、SEPP1、SERPING1、SIX1、SLC16A7、STOM、SVIL、SYNE3、TENM2、TIMP3、TIMP4、TJP2、TM4SF1、TMEM140、VIM、ZBED3、ZEB2、LINC00341、AKR1C1 |

**Supplementary Table 1.** 98 upregulated genes and 66 downregulated genes.
